# Coordination Behavior of Ni^2+^, Cu^2+^, and Zn^2+^ in Tetrahedral 1-Methylimidazole Complexes: A DFT/CSD Study

**DOI:** 10.1155/2018/3157969

**Published:** 2018-05-14

**Authors:** Samuel Tetteh

**Affiliations:** Department of Chemistry, School of Physical Sciences, College of Agriculture and Natural Sciences, University of Cape Coast, Cape Coast, Ghana

## Abstract

The interaction between nickel (Ni^2+^), copper (Cu^2+^), and zinc (Zn^2+^) ions and 1-methylimidazole has been studied by exploring the geometries of eleven crystal structures in the Cambridge Structural Database (CSD). The coordination behavior of the respective ions was further investigated by means of density functional theory (DFT) methods. The gas-phase complexes were fully optimized using B3LYP/GENECP functionals with 6-31G^∗^ and LANL2DZ basis sets. The Ni^2+^ and Cu^2+^ complexes show distorted tetrahedral geometries around the central ions, with Zn^2+^ being a perfect tetrahedron. Natural bond orbital (NBO) analysis and natural population analysis (NPA) show substantial reduction in the formal charge on the respective ions. The interaction between metal d-orbitals (donor) and ligand orbitals (acceptor) was also explored using second-order perturbation of the Fock matrix. These interactions followed the order Ni^2+^ > Cu^2+^ > Zn^2+^ with Zn^2+^ having the least interaction with the ligand orbitals. Examination of the frontier orbitals shows the stability of the complexes in the order Ni^2+^ > Cu^2+^ < Zn^2+^ which is consistent with the Irving–Williams series.

## 1. Introduction

Metalloproteins play important roles in the structure and physiology of cells. They account for nearly half of all proteins in nature [1]. Some of their cellular functions include water oxidation, photosynthesis, respiration, molecular oxygen reduction, and zinc fingers [1, 2]. The binding stability of divalent transition metals in metalloproteins has been largely studied in terms of semiempirical and qualitative theories such as the hard and soft acid and base principles of Parr and Pearson and the Irving–Williams series of stability constants [3]. Yet the fundamental role of metal ions in the structure and function of metalloproteins is still a matter of ongoing research [4, 5]. The amino acid histidine is a common ligand in metalloproteins [6]. The imidazole ring of histidine is an important five-membered heterocycle that is widely present in natural products and most synthetic molecules. Because of its unique electronic structure, imidazole-based compounds are widely used as anticancer, antifungal, antibacterial, anti-inflammatory, antihistaminic, and other medicinal agents [7–10]. It readily binds with a variety of enzymes and receptors in biological systems through hydrogen bonds, coordinate covalent bonds, ion-dipole interactions, *π*-*π*^∗^ interactions, and van der Waals forces, thereby exhibiting broad bioactivities [7].

Imidazole is largely considered an *N*-donor in most of its coordination complexes [11]. This interaction largely affects the formal charge of the coordinating electrophile. According to Hasegawa et al., the total charge of 4-methylimidazole increased upon binding to Zn^2+^, indicating that some negative charge is transferred to the Zn^2+^ in the complex. This charge delocalization has a tendency of reducing the formal charge on Zn^2+^ to 1.70. Similar atomic charges were observed in the nitrogen atoms of the 4-methylimidazole ligand [12]. This charge delocalization could greatly affect the reactivity and catalytic activity of imidazole-based transition metal complexes.

A study by Rulíšekr and Vondrášek on the coordination geometries of selected metal ions in metalloproteins with data from the Protein Data Bank suggests that Ni^2+^, Cu^2+^, and Zn^2+^ tend to bind in tetrahedral coordination modes although there are other geometries such as octahedral and square planar [3]. A search in the Cambridge Structural Database version 5.38 (November 2016) plus one update revealed one Ni complex bearing 1-methylimidazole ligand [13]. In this complex, the nickel ion is octahedrally coordinated with the trien ligand bonded through its four nitrogen atoms with the remaining two coordination sites occupied by 1-methylimidazole ligands. Ten copper complexes bearing 1-methylimidazole ligands were also found. These include molecules with the following reference codes: BEJGUS [14], CAHJAW [15], and CUSHON [16] with square-planar coordination around the copper ion through two carboxylic oxygen atoms and two 1-methylimidazole nitrogen atoms; MACCUA10 [17] of square-pyramidal geometry with the four nitrogen atoms of the macrocycle forming the basal plane and 1-methylimidazole nitrogen at the apex; GALLAG [18] of square-planar coordination through four 1-methylimidazole ligands; GALLOU [18], a tetragonally distorted octahedral complex with two water molecules *trans* to each other and four 1-methylimidazole ligands in the square plane; KAYPEF [19] (chloro(glycinato)(1-methylimidazole)copper(II) complex) with a square-pyramidal structure having four close ligating atoms (N2OCl) and an axial chlorine ligand; CEZLOI [20], a Cu^+^ complex with S_4_ site symmetry in which the Cu ion is tetrahedrally coordinated with four 1-methylimidazole ligands; and finally, the complexes GALLEK and GALLIO with tetrahedral geometries in which the copper ions are linked to a central oxygen atom with *μ*_2_-Cl atoms above each edge of the tetrahedron and terminal 1-methylimidazole ligand. However, there was no zinc complex bearing 1-methylimidazole ligand at the time of preparing this manuscript. Considering the importance of nickel, copper, and zinc ions in the structure and function of metalloproteins, this article investigates the effect of bonding on the energies of the d-orbitals of Ni^2+^, Cu^2+^, and Zn^2+^ in tetrahedral ligand fields. It also reports the effect on the formal charges of the ions upon coordination to the 1-methylimidazole (1-MeIm) ligand.

## 2. Methodology

### 2.1. CSD Analysis

The ligand and transition metal complexes were analyzed with version 5.38 of the CSD (November 2016) plus one update. The CSD program ConQuest Version 1.19 was used to perform substructure searches of Ni, Cu, and Zn complexes bearing 1-methylimidazole ligands. The accepted entries met the following criteria: 3D coordinates determined; crystallographic R factor ≤ 0.05; no disorder in the crystal structure; no errors in the structure; no polymeric bonding; no ions; and no powder structures and only organometallic structures (according to standard CSD definitions) [21]. The search revealed eleven crystal structures: one nickel and ten copper complexes. No zinc complex was found in the database. The geometry of *N*-methylimidazole in a novel 4- and 5-coordinated silicon complex (reference code: GAGXER) [22] was used as the reference. The 3D structures of all the complexes were visualized using version 3.9 of the CSD program *Mercury* [23].

### 2.2. Computational Studies

**Figure 1:**
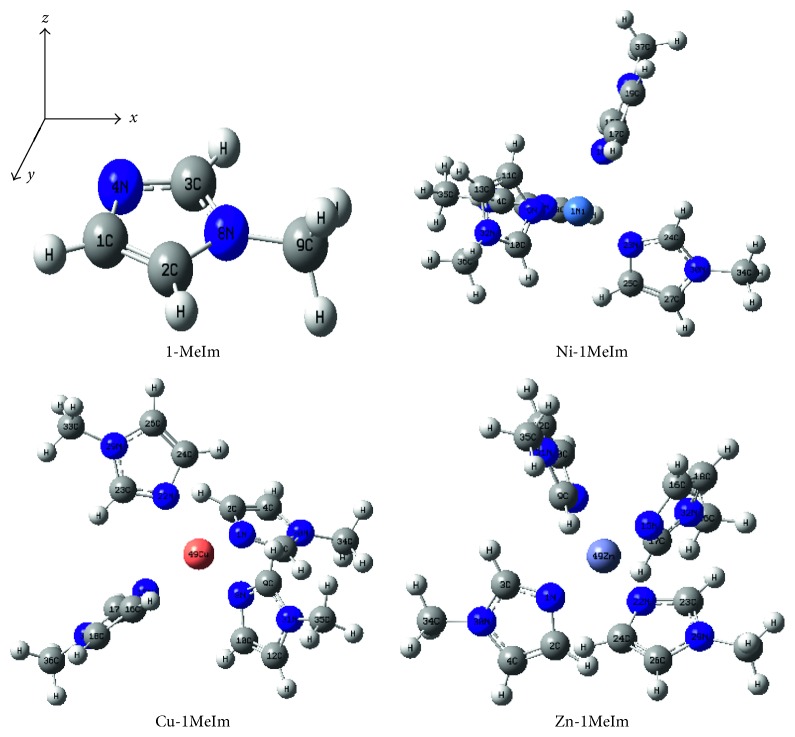
Optimized geometries of 1-methylimidazole and the respective complexes.

All input files were prepared using the GaussView 5.0.8 molecular structure viewer [24]. The Gaussian 09 program was used to perform all the computations. All structures were optimized with the density functional theory (DFT) using B3LYP three-parameter hybrid functionals with no constraints in the respective geometries. Effective core potentials (ECPs) were used to represent the valence electrons of Ni^2+^, Cu^2+^, and Zn^2+^ and the basis set of double-*ζ* quality associated with pseudopotentials known as LANL2DZ [25]. Because of the size of the complexes and the accompanying computational cost, the 6-31G^∗^ basis set was used for all other atoms. Similar basis sets have been used to study the geometries and molecular orbitals of transition metal complexes [26]. Frequency analyses were employed to confirm that the optimized geometries were at stationary points corresponding to local minima with no imaginary frequencies [21]. The optimized geometries with the accompanying numbering scheme and the Cartesian atomic coordinates are shown in Figure 1.

## 3. Results and Discussion

### 3.1. Geometry Optimization

Figure 1 shows the gas-phase optimized geometries and the selected atomic numbering scheme of 1-methylimidazole and the respective complexes. It also indicates the Cartesian atomic coordinates. Selected bond lengths of the optimized structures are shown in Table 1. A search in the version 5.38 of the CSD (November 2016) showed that there was no crystal structure of 1-methylimidazole in the database. However, the geometry of *N*-methylimidazole in a novel 4- and 5-coordinated silicon complex (reference code: GAGXER) [22] was used as the reference. The bond lengths did not differ significantly from that of the optimized structure. The slight differences could be attributed to the fact that the optimization was carried out on a single molecule in the gas phase with no intermolecular interactions, whereas in the crystal structure, there are lattice interactions which affect the bond parameters [21].

Generally, the intramolecular bond lengths in 1-MeIm were not affected by the coordination. Notable exceptions include the C1-N4, C2-N8, and C3-N8 bonds which recorded slight decreases in bond lengths as a result of coordination to the metal ion. The metal-ligand bond lengths (M-N) ranged between 2.018 and 2.036 Å in the Ni-1MeIm complex and between 2.017 and 2.038 Å in the Cu-1MeIm analogue but remained the same (2.085 Å) in the zinc complex. The differences in the Ni-N and Cu-N bond lengths could be attributed to Jahn–Teller distortions for the (e)^4^(t_2_)^4^ and (e)^4^(t_2_)^5^ electronic configurations in the respective Ni-1MeIm and Cu-1MeIm complexes [27]. Zn^2+^ has fully occupied d-orbitals and interacts weakly with ligand orbitals and maintains a spherical isotropy. The C3-H7 bond length did not show much variation as a result of the bonding interaction of the ligand with the metal ions. The observed differences in bond lengths were further investigated by a series of density functional theory (DFT) calculations.

### 3.2. Natural Bond Orbital (NBO) Analysis and Charge Distribution

The effect of coordination and electron distribution on the ligands and metal d-orbitals was assessed by the NBO and second-order perturbation of the Fock matrix. This provides details about the type of hybridization, nature of bonding, and strength of interaction between the metal ion and ligand [28].  Table 2 shows the occupancy and electron density (ED) of molecular orbitals in 1-MeIm. The C1-N4 *σ*-bond in the uncoordinated ligand has an electron density of 1.980 and an occupancy of 41.4% C1 and 58.6% N4. Upon coordination, the electron density reduced to 0.990 in both Ni-1MeIm and Cu-1MeIm with the occupancies also changing to 38.1% and 61.9% for C1 and N4, respectively.

The reduced electron density in the C1-N4 *σ*-bond could have been delocalized onto the metal ions [29] to reduce their formal charges from +2 to −0.2985 and +0.2132, respectively, as shown in Table 3. Despite the fact that occupancy changed to 38.8% C1 and 61.7% N4 in Zn-1MeIm, the ED was not affected. Similar effects were observed in the C3-N4 *σ*-bond. The C3-N4 *π*-bond observed in the free ligand was, however, nonexistent in the Ni^2+^ and Cu^2+^ complexes. These *π*-electrons could have contributed to reducing the strength of the formal charges on the respective metal ions as observed in Table 3. Generally, although there were substantial changes in the occupancy of the bonds in the 1-MeIm upon coordination to the Zn^2+^ ion, the ED on the ligand remained unchanged as compared to the free ligand.

From Table 3, the calculated formal charges on the central metal ions have been reduced to −0.2985, +0.2132, and +1.3850, respectively. This reduction in the ionic charge is higher in Ni-1MeIm and Cu-1MeIm than in Zn-1MeIm. As shown in Table 2, there is higher delocalization of ligand electrons onto Ni^2+^ and Cu^2+^ than in the case of Zn^2+^. The natural charge on the N4 is −0.4842 in the free ligand but −0.03501 and −0.3524 in the Ni^2+^ and Cu^2+^ complexes, respectively. It however increased to −0.6791 in the Zn^2+^ complex.

Similarly, natural charges on C1, C2, C3, N8, and C9 which decreased in the Ni-1MeIm and Cu-1MeIm complexes, however, increased in the Zn^2+^ analogue. These support the fact that although there is substantial delocalization of ligand-bonding electrons onto Ni^2+^ and Cu^2+^ d-orbitals [30], these electrons are, however, localized on the ligand ring of the Zn-1MeIm complex, making it more nucleophilic.

The 3d-orbital occupancy and energies of the complexes are assessed in Table 4. It is shown that the *t*_2_ orbitals (*d*_*xy*_, *d*_*xz*_, and *d*_*xy*_) of Ni-1MeIm have higher energies (−0.611 eV), with the *e* orbitals (*d*_*x*^2^−*y*^2^_ and *d*_*z*^2^_) having lower energy (−0.626 eV). This is typical of metal d-orbitals in a tetrahedral field [27].

There is, however, a reduced occupancy (≈0.9950) in all the orbitals. These electrons could be delocalized onto antibonding or non-Lewis type (Rydberg) molecular orbitals [31]. Similar occupancies were observed in the Cu^2+^ ion. With regard to the energy levels, the *d*_*xz*_ and *d*_*yz*_ orbitals were degenerated (−0.612 eV), but the *d*_*xy*_ orbital sunk low in energy (−0.660 eV) below the *e* energy levels. The *d*_*x*^2^−*y*^2^_ orbital was also lower in energy than the *d*_*z*^2^_ orbital. These differences in the energy support the Jahn–Teller distortions observed in Table 1 as a result of unequal occupancy of the *t*_2_ orbitals in a tetrahedral field. Similarly, the Cu-1MeIm bond lengths also showed variations as observed in Table 1. In the case of Zn-1MeIm, the d-orbital occupancy was the same (≈2), showing that the d-electrons were localized within the Zn^2+^ d-orbitals [12]. Furthermore, the d-orbitals were degenerated with no d-d splitting. This explains the uniformity in the Zn-1MeIm bond lengths observed in Table 1.

To further probe the low occupancy of the Ni^2+^ and Cu^2+^ d-orbitals, second-order perturbation analysis of the Fock matrix was performed to investigate the interaction between metal d-orbitals (donor) and non-Lewis orbitals (acceptor) on the coordinating atom of the ligands. Tables 5–7 summarize the interaction between the d-electrons on the metal ions and the acceptor sites on the respective ligands. The highest energies, E(2), were recorded for all the complexes.

On the contrary, weaker interactions were observed in the case of Zn-1MeIm. Notable interactions include donation of LP(5) on Zn49 to RY ∗ (2)N1, RY ∗ (2)N8, RY ∗ (2)N15, and RY ∗ (2)N22 of energies 0.11, 0.10, 0.10, and 0.10 kcal/mol, respectively.

These interaction energies are, however, lower than those observed in the Ni-1MeIm and Cu-1MeIm. This explains the higher occupancies of the Zn^2+^ d-orbitals compared with those of the Ni^2+^ and Cu^2+^ analogues as shown in Table 4.

The energies of the frontier orbitals are important in describing the chemical properties of molecules [32]. The energy of the highest occupied molecular orbital (HOMO) gives an indication of the electron-donating ability of the complex [33].

**Figure 2:**
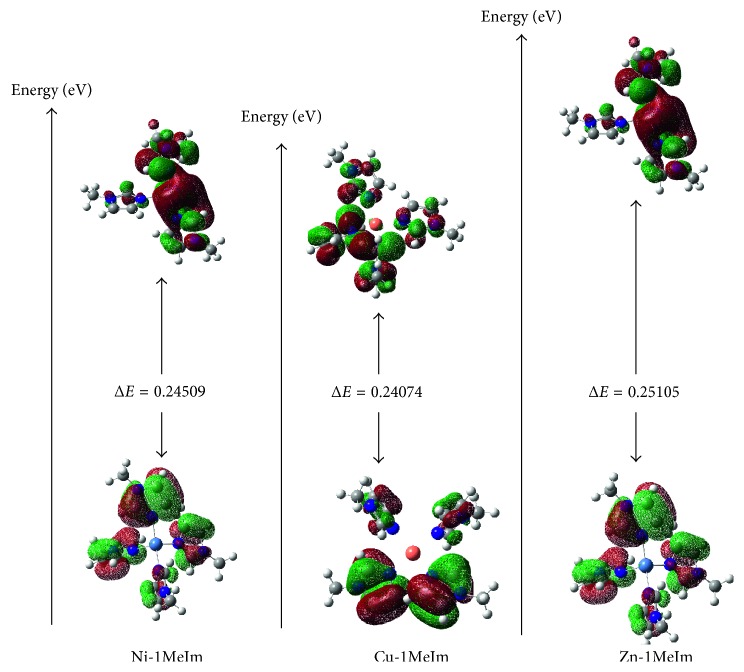
Frontier orbital energies of the complexes.

The higher the energy of the HOMO, the easier it is to donate electrons into the lowest unoccupied molecular orbital (LUMO). Also, the larger the energy difference between these frontier orbitals, the more stable the complex.  Figure 2 shows the energy gap between the frontier orbitals in the respective complexes. The *E*_HOMO_ − *E*_LUMO_ gap is in the order Ni-1MeIm > Cu-1MeIm < Zn-1MeIm. This order is in agreement with the Irving–Williams series [27].

## 4. Conclusion

This article assessed the coordination behavior of Ni^2+^, Cu^2+^, and Zn^2+^ ions in tetrahedral 1-methylimidazole ligand fields. Measurement of the bond lengths of the optimized structures revealed Jahn–Teller distortions in the Ni-1MeIm and Cu-1MeIm complexes. These distortions were, however, absent in Zn-1MeIm. A natural bond orbital (NBO) analysis of the molecular orbitals shows substantial decrease in the formal charges on the metal ions upon coordination to the ligands. This was in the order Ni^2+^ ˂ Cu^2+^ ˂ Zn^2+^ with Zn^2+^ being the most electropositive one. Second-order perturbation of the Fock matrix shows higher interaction energies between Ni^2+^ 3d-orbitals (donor) and ligand orbitals (acceptor) than in the case of Zn^2+^ 3d-orbitals (donor). Finally, the frontier orbitals were assessed as a measure of the stability of the complexes. The energy of the *E*_HOMO_ − *E*_LUMO_ gap was in the order Ni-1MeIm > Cu-1MeIm < Zn-1MeIm which is consistent with the Irving–Williams series.

## Figures and Tables

**Table 1:** Selected bond lengths of the optimized structures.

Bond	Length (Å)				
	1-MeIm		Ni-1MeIm	Cu-1MeIm	Zn-1MeIm
	Experimental	Calculated			
C1-C2	1.312	1.380	1.367	1.367	1.367
C1-N4	1.327	1.396	1.385	1.386	1.387
C1-H5	0.946	1.077	1.079	1.079	1.080
C2-H6	0.957	1.078	1.080	1.080	1.080
C2-N8	1.349	1.393	1.382	1.381	1.381
C3-N4	1.301	1.333	1.335	1.335	1.335
C3-H7	1.031	1.078	1.080	1.079	1.081
C3-N8	1.348	1.381	1.347	1.346	1.367
C9-N8	1.474	1.461	1.467	1.467	1.467
C9-H10	0.919	1.095	1.092	1.092	1.092
C9-H11	0.995	1.095	1.092	1.092	1.092
C9-H12	1.219	1.093	1.091	1.091	1.091
M-N1	—	—	2.018	2.038	2.085
M-N2	—	—	2.036	2.018	2.085
M-N3	—	—	2.032	2.017	2.085
M-N4	—	—	2.027	2.038	2.085

M-N represents the metal-nitrogen bond.

**Table 2:** NBO population analysis for the respective compounds.

Bond	Type	1-Methylimidazole		Ni-1MeIm		Cu-1MeIm		Zn-1MeIm	
		ED	Occupancy	ED	Occupancy	ED	Occupancy	ED	Occupancy
C1-C2	*σ*	1.986	48.8% C1-51.2% C2	0.992	49.6% C1-50.4% C2	0.992	49.6% C1-50.4% C2	1.982	49.6% C1-50.4% C2
C1-C2	*π*	1.861	48.5% C1-51.5% C2	0.921	50.1% C1-49.9% C2	0.921	50.0% C1-50.0% C2	1.843	50.3% C1-49.7% C2
C1-N4	*σ*	1.980	41.4% C1-58.6% N4	0.990	38.1% C1-61.9% N4	0.990	38.1% C1-61.9% N4	1.981	38.3% C1-61.7% N4
C1-H5	*σ*	1.986	62.7% C1-37.3% H5	0.992	62.9% C1-37.1% H5	0.992	63.0% C1-37.0% H5	1.985	62.7% C1-37.3% H5
C2-H6	*σ*	1.986	62.6% C2-37.4% H6	0.993	63.5% C2-36.5% H6	0.992	63.6% C2-36.4% H6	1.985	63.5% C2-36.5% H6
C2-N8	*σ*	1.984	36.0% C2-64.0% N8	0.991	36.1% C2-63.9% N8	0.991	36.0% C2-64.0% N8	1.982	36.1% C2-63.9% N8
C3-N4	*σ*	1.987	41.9% C3-58.1% N4	0.991	38.9% C3-61.1% N4	0.992	38.8% C3-61.2% N4	1.985	39.1% C3-60.9% N4
C3-N4	*π*	1.871	43.5% C3-56.5% N4	—	—	—	—	1.891	34.7% C3-65.3% N4
C3-H7	*σ*	1.985	62.2% C3-37.8% H7	0.992	62.6% C3-37.4% H7	0.992	62.5% C3-36.8% H7	1.984	62.5% C3-37.5% H7
C3-N8	*σ*	1.989	35.6% C3-64.4% N8	0.993	36.4% C3-63.6% N8	0.993	36.4% C3-63.6% N8	1.985	36.4% C3-63.6% N8
C9-N8	*σ*	1.993	36.4% C9-63.6% N8	0.995	34.8% C9-65.2% N8	0.995	34.8% C9-65.2% N8	1.990	34.8% C9-65.2% N8
C9-H10	*σ*	1.989	62.8% C9-37.2% H10	0.995	63.2% C9-36.8% H12	0.995	63.2% C9-36.8% H12	1.990	62.6% C9-37.4% H10
C9-H11	*σ*	1.989	62.8% C9-37.2% H11	0.993	63.2% C9-36.8% H11	0.993	63.2% C9-36.8% H11	1.987	63.2% C9-36.8% H11
C9-H12	*σ*	1.990	62.7% C9-37.3% H12	0.993	62.6% C9-37.4% H10	0.993	62.6% C9-37.4% H10	1.987	63.2% C9-36.8% H12

**Table 3:** NPA atomic charge distributions of some selected atoms in the complexes and free ligand.

Atom	Charge			
	1-MeIm	Ni-1MeIm	Cu-1MeIm	Zn-1MeIm
C1	−0.1277	−0.0356	−0.0338	−0.0741
C2	−0.1175	−0.0300	−0.0289	−0.0559
C3	+0.1396	+0.1241	+0.1264	+0.2410
N4	−0.4842	−0.3501	−0.3524	−0.6791
H5	+0.2505	+0.1271	+0.1282	+0.2523
H6	+0.2488	+0.1347	+0.1350	+0.2697
H7	+0.2395	+0.1236	+0.1226	+0.2457
N8	−0.4123	−0.1783	−0.1771	−0.3545
C9	−0.4957	−0.1789	−0.2357	−0.4711
H10	+0.2520	+0.1318	+0.1316	+0.2630
H11	+0.2520	+0.1312	+0.1318	+0.2626
H12	+0.2552	+0.1270	+0.2132	+0.2542
Ni^2+^	—	−0.2985	—	—
Cu^2+^	—	—	+0.2132	—
Zn^2+^	—	—	—	+1.3850

**Table 4:** 3d-orbital occupancy and energy of the complexes.

Orbital	Ni-1MeIm		Cu-1MeIm		Zn-1MeIm	
	Occupancy	Energy (eV)	Occupancy	Energy (eV)	Occupancy	Energy (eV)
*d*_*xz*_	0.9945	−0.609	0.9963	−0.612	1.9937	−0.817
*d*_*xy*_	0.9950	−0.611	0.9958	−0.660	1.9962	−0.818
*d*_*yz*_	0.9964	−0.611	0.9963	−0.612	1.9937	−0.817
*d*_*x*^2^−*y*^2^_	0.9939	−0.626	0.9968	−0.628	1.9936	−0.818
*d*_*z*^2^_	0.9942	−0.623	0.9923	−0.605	1.9956	−0.818

**Table 5:** Second-order perturbation theory of the Fock matrix in the NBO basis for Ni-1MeIm.

Donor	Acceptor							
Ni1	N2	E(2)	N9	E(2)	N16	E(2)	N23	E(2)
LP(1)	RY ∗ (1)	0.24	RY ∗ (1)	0.03	RY ∗ (2)	0.16	RY ∗ (1)	0.24
LP(2)	RY ∗ (1)	0.27	RY ∗ (2)	0.03	RY ∗ (1)	0.45	RY ∗ (2)	0.08
LP(3)	RY ∗ (2)	0.13	RY ∗ (3)	0.05	RY ∗ (1)	0.10	RY ∗ (1)	0.13
LP(4)	RY ∗ (1)	0.07	RY ∗ (1)	0.07	RY ∗ (1)	0.07	RY ∗ (1)	0.68
LP(5)	RY ∗ (2)	0.04	RY ∗ (1)	0.32	—	—	RY ∗ (3)	0.06

Notable interactions in Ni-1MeIm include LP(2)Ni1 → RY ∗ (1)N2 and LP(2)Ni1 → RY ∗ (1)N16 of energies 0.27 and 0.45 kcal/mol, respectively, and also LP(4)Ni1 → RY ∗ (1)N23 of energy 0.68 kcal/mol. This delocalization of d-electrons onto non-Lewis orbitals could contribute to the decrease in occupancy of the Ni^2+^ d-orbitals [31]. In the case of Cu-1MeIm, similar interaction energies were observed: LP(1)Cu49 → RY∗(1)N1, LP(1)Cu49 → RY ∗ (1)N22, LP(4)Cu49 → RY ∗ (1)N1, and LP(4)Cu49 → RY ∗ (1)N22 of energies 0.32, 0.32, 0.29, and 0.29 kcal/mol, respectively.

**Table 6:** Second-order perturbation theory of the Fock matrix in the NBO basis for Cu-1MeIm.

Donor	Acceptor							
Cu49	N1	E(2)	N8	E(2)	N15	E(2)	N22	E(2)
LP(1)	RY ∗ (1)	0.32	RY ∗ (1)	0.19	RY ∗ (1)	0.19	RY ∗ (1)	0.32
LP(2)	—	—	RY ∗ (1)	0.11	RY ∗ (1)	0.09	—	—
LP(2)	—	—	RY ∗ (2)	0.04	RY ∗ (2)	0.03	—	—
LP(3)	—	—	RY ∗ (1)	0.07	—	—	—	—
LP(4)	RY ∗ (1)	0.29	RY ∗ (1)	0.23	RY ∗ (1)	0.22	RY ∗ (1)	0.29
LP(4)	RY ∗ (5)	0.07	RY ∗ (5)	0.05	RY ∗ (5)	0.05	RY ∗ (5)	0.06
LP(5)	RY ∗ (2)	0.07	—	—	—	—	RY ∗ (2)	0.07

**Table 7:** Second-order perturbation theory of the Fock matrix in the NBO basis for Zn-1MeIm.

Donor	Acceptor							
Zn49	N1	E(2)	N8	E(2)	N15	E(2)	N22	E(2)
LP(4)	RY ∗ (2)	0.07	RY ∗ (2)	0.07	RY ∗ (2)	0.07	RY ∗ (2)	0.07
LP(4)	RY ∗ (3)	0.09	RY ∗ (3)	0.09	RY ∗ (3)	0.09	RY ∗ (3)	0.09
LP(5)	RY ∗ (2)	0.11	RY ∗ (2)	0.10	RY ∗ (2)	0.10	RY ∗ (2)	0.10

## Data Availability

The data used to support the findings of this study are available from the corresponding author upon request.
